# Coinfection with HIV-1 skews iNKT cells toward TCR anergy and limited expansion potential in people with hepatitis C

**DOI:** 10.3389/fimmu.2025.1469473

**Published:** 2025-04-22

**Authors:** Danielle Nettere, Scott White, Grant Williams, Shalini Jha, M. Anthony Moody, Cliburn Chan, Guido Ferrari, Susanna Naggie

**Affiliations:** ^1^ Department of Surgery, Duke University School of Medicine, Durham, NC, United States; ^2^ Molecular Genetics and Microbiology, Duke University School of Medicine, Durham, NC, United States; ^3^ Center for Human Systems Immunology, Duke University School of Medicine, Durham, NC, United States; ^4^ Duke University, Durham, NC, United States; ^5^ Department of Pediatrics, Duke University School of Medicine, Durham, NC, United States; ^6^ Department of Medicine, Duke University School of Medicine, Durham, NC, United States

**Keywords:** HIV, iNKT, HCV, HIV/HCV coinfection, flow cytometry

## Abstract

**Introduction:**

Hepatitis C virus (HCV) infection remains a leading cause of morbidity and mortality in people with human immunodeficiency virus (HIV). Liver fibrosis progression is more rapid in people with HIV/HCV coinfection compared to HCV monoinfection and the rate of resolution of liver fibrosis after HCV cure is unknown in people with HIV. Invariant natural killer T (iNKT) cells are enriched in the liver and play important roles in initiating immune responses to hepatotropic pathogens and promoting healing following injury. It was recently reported that the pro-healing CD4+ iNKT cells are preferentially infected and depleted in early HIV infection, but this effect on HCV-related liver disease outcomes is unclear.

**Methods:**

Here we examined and compared peripheral blood iNKT cells from people with HIV/HCV coinfection and people with HIV and HCV monoinfection or no infection (controls). We evaluated the iNKT cells’ expansion potential and phenotype using an unbiased Uniform Manifold Approximation and Projection (UMAP) and clustering based approach.

**Results:**

We observed that circulating iNKT cells from people with HIV and HIV/HCV coinfection have impaired expansion to T-cell receptor (TCR) stimulation. We also observed an enrichment of the CD8+ and CD57+ iNKT subsets, which are thought to represent terminally differentiated iNKT cells. HCV monoinfection on the other hand minimally impacted iNKT phenotypes compared to controls.

**Discussion:**

The changes observed in iNKT phenotype and proliferative ability in people with HIV/HCV coinfection suggest an impairment that may be contributing to the enhanced pathogenesis during coinfection and could inform novel therapeutic approaches.

## Introduction

Invariant natural killer T (iNKT) cells represent a low-frequency type of alpha-beta T cell that can robustly respond to pathogens without initial priming (1). iNKT cells have a fixed V(D)J rearrangement of Vα24-Jα18 in human with restricted β-chain usage, most commonly Vβ11 (2). This T-cell receptor (TCR) does not recognize peptides presented in major histocompatibility complex (MHC) I or II but rather recognizes glycolipids presented in CD1d, an MHC-like molecule expressed by hepatocytes and several other antigen-presenting cells (3, 4). In humans, the frequency of iNKT cells among circulating lymphocytes is small, ranging from 0.1% to 0.5%, whereas their frequency is 10-fold higher in the liver (range 1%–5%) (5). Their enrichment in tissues, especially the liver, places them at an important interface for liver-associated pathogens. In response to pathogens and lipid dysregulation, iNKT cells can rapidly recognize glycolipids in CD1d and generate a cytokine-mediated cytotoxic response. The differentiation stages of iNKT cells can be defined based on the expression of CD4 and CD8 markers. CD4+/CD8− iNKT cells represent a naïve/early subset, double-negative iNKT cells are a more mature effector cell, and CD4−/CD8+ are thought to be terminally differentiated effectors (6). The CD4+ iNKT cells are thought to recruit monocytes via IL-4 and IL-13 secretion for liver repair following injury, whereas the CD8+ iNKT cells are thought to participate more in proinflammatory and antipathogen responses, although these divisions are not fully established (7, 8).

Our understanding of the role of iNKT in human disease is rather limited because of the low circulating frequency in the periphery, which often requires the collection of large volumes of blood to meaningfully evaluate this population. iNKT cells are known to expand in acute hepatitis C virus (HCV) infection, and humanized mouse models have suggested that they play a protective role in acute HCV infection (9–12). However, they have also been implicated in promoting the pathogenesis of several fibrinogenic liver diseases including metabolic dysfunction-associated steatohepatitis (MASH) and metabolic dysfunction-associated steatotic liver disease (MASLD) through chronic inflammation (5, 13–17). CD4+ iNKT cells are highly susceptible to human immunodeficiency virus (HIV) infection and are rapidly depleted in acute infection (18–21); however, it is unknown to what degree antiretroviral therapy (ART) aids their restoration.

Liver disease, especially viral-associated liver disease, is a major cause of morbidity and mortality in people with HIV (PWH) (22). PWH have worse liver-related outcomes with HCV infection compared to those without HIV, even with viral suppression on ART (23, 24). We hypothesized that HIV infection alters both the circulating and tissue-resident iNKT compartment and may enhance HCV pathogenesis. To address a fundamental question of how iNKT cells are impacted by HIV and HCV viral infections, with particular interest in the role of coinfection, we collected peripheral blood samples from people with HIV, HCV, and HIV/HCV and assessed the phenotype of these cells and their ability to expand in response to stimulation with their cognate ligand. We performed our analyses using an unbiased Uniform Manifold Approximation and Projection (UMAP)-based approach for various iNKT surface markers that reflect their activation, maturation, and terminal differentiation. We observed that HIV and HIV/HCV coinfection was associated with major shifts in the circulating iNKT compartment and decreased the ability of these cells to expand to stimulation. We concluded that HIV infection significantly alters the circulating iNKT compartment toward TCR downregulation, terminal differentiation, and replicative senescence. We believe that this may reflect the ability of tissue-resident iNKT cells to engage and respond to HCV-infected hepatocytes and promote healing following injury.

## Materials and methods

### Participant information

This is a prospective cohort study in which adult patients (≥18 years of age) with HIV/HCV coinfection and HIV or HCV monoinfection were identified from the Duke Infectious Diseases Clinic and approached for interest in participating in the study and, if interested, completed an informed consent process. Volunteers without HIV or HCV were also recruited through the Duke Clinical Research Volunteer Registry to serve as a control population. The proposed research was approved by the Duke Institutional Review Board.

The study population inclusion criteria included CD4 > 350 cells/mL and HIV viral suppression on ART for >1 year for the people with HIV infection and evidence of HCV viremia with either qualitative or quantitative HCV RNA PCR documented in the electronic health record and no history of HCV cure for people with HCV infection. Exclusion criteria included active hepatitis B virus (HBV) infection (defined as detectable HBV surface antigen), other active viral infection, and receipt of any medication that alters the immune system including chemotherapy, immunosuppressants, and immunomodulatory agents.

After the provision of informed consent, participants’ whole blood was collected by venipuncture, and minimal clinical information was collected including demographics, most recent CD4 count and HIV viral load, and ART regimen.

### Cell preparation and stimulation

Peripheral blood mononuclear cells (PBMCs) were isolated from whole blood, processed using standard Ficoll gradient separation, and cryopreserved in liquid nitrogen within 8 hours from collection. Cells were thawed with warmed Roswell Park Memorial Institute (RPMI) supplemented with 10% Fetal Bovine Serum (FBS) and penicillin, streptomycin, gentamicin, and l-glutamine (complete media) and resuspended at 10 (6)/mL. Cells were rested overnight in complete media (resting), or stimulated for 2 weeks with 50 U/mL IL-2 (unstimulated) or 2 weeks with 50 U/mL IL-2 and 100 ng/mL of alpha-galactosylceramide (alpha-gal), and cultured for 2 weeks at 37°C and 5% CO_2_. The culture medium was replaced every 3–4 days for the duration of the experiments.

### Flow cytometry staining and acquisition

After either overnight or 2 weeks of incubation, cells were resuspended and transferred to 5-mL Fluorescence activated cell sorting (FACS) tubes. Cells were pelleted at 500 g for 5 minutes and then washed with Dulbecco's Phosphate-Buffered Saline (DPBS) twice before viability staining with a commercially available viability dye (LIVE/DEAD Fixable Aqua Dead Cell Stain 1:800 dilution; Invitrogen, Carlsbad, CA, USA; cat L34957). After two washes with DPBS with 1% FBS, the cells were stained for 30 minutes at room temperature with the following commercially available antibodies and CD1d dextramer: CD57 BV421 (BD, San Jose, CA, USA; cat 564896), CD4 BV605 (BioLegend, San Diego, CA, USA; cat 317438), CD8 BV650 (BioLegend; cat 201041), Vβ11 FITC (BioLegend; cat 125905), CD1d phycoerythrin (PE) (Immudex, Copenhagen, Denmark; cat XD8002), CD45Ro PE-TR (Beckman Coulter, Brea, CA, USA; cat IM2712U), HLA-DR PerCP Cy5.5 (BioLegend; cat 307629), CD27 PeCy5 (eBioscience, San Diego, CA, USA; cat 15-0279-42), Vα24 PeCy7 (Beckman Coulter; cat A66907), CD38 AF700 (BioLegend; cat 303524), Vα24-Jα18 APC (BioLegend; cat 342908), and CD3 APC-H7 (BD; cat 560176). Cells were then washed twice with DPBS with 1% FBS, fixed with 1% paraformaldehyde, and acquired using LSRII Fortessa within 24 hours. Flow cytometry gates were identified using concatenated sample files, and phenotype analysis was conducted on populations with 30 events or greater.

### FlowKit and statistical analysis

Sample data were compensated and preprocessed in FlowJo (10.10.0), and events in the iNKT gate were exported for downstream processing as FCS files. FCS files were read using the FlowKit Python package (25), and event data were combined into a single data frame with columns for groups (HCV, HIV, or HIV/HCV), stimulation conditions (resting, unstimulated, and alpha-gal), and scatter and fluorescent channels. Controls were not used for UMAP projection due to a multiyear delay in collection to prevent a batch effect. For visualization, UMAP (26) was used for dimension reduction using the UMAP-learn package. Spectral clustering on scaled event data was performed using the Leiden algorithm (27) as implemented in the PhenoGraph package (28). For reproducibility, a Jupyter notebook and the data necessary to replicate FlowKit-generated figures in the manuscript were held in the Ferrari-Pollara server according to Duke institutional requirements and can be accessed upon request from the administrators. Mean differences in surface expression of markers and frequency were compared using paired Mann–Whitney U tests with a Bonferroni correction.

## Results

### Study population

Twenty-six participants (HIV/HCV, n = 7; HIV, n = 7; HCV, n = 6; control, n = 6) provided consent and were enrolled in this prospective cohort study. The mean age was 51 (42–60) with 53.8% (14/26) male and 84.6% (22/26) identified as Black/African American (**Table 1**). For the people with HIV and HIV/HCV, the mean CD4 was 746.7 (SD = 287), and ART regimens included 100% nucleoside reverse transcriptase inhibitors (NRTIs), 7.1% non-nucleoside reverse transcriptase inhibitors (NNRTIs), 35.7% protease inhibitors (PIs), 35.7% integrase inhibitors (IIs), and 57.1% cocktail. The median fibrosis (FIB)-4 for people with HCV and HIV/HCV was 2.08 (1.05–2.72) and 2.98 (1.36–8.58), respectively.

**Table 1:** Patient characteristics.

	HIV	HCV	HIV/HCV	Control
Number of participants (N)	7	6	7	6
Mean Age (range)	53 (42–59)	53 (45–60)	52 (45–60)	46 (42–51)
% Male	85.7%	33.3%	57.1%	33.3%
% Black	85.7%	66.6%	100%	83.3%
HIV VL < 20 c/mL	100%	-	100%	-
CD4 count	801.4 (530–1,274)	-	692 (413–1,264)	-
FIB-4	-	2.08 (1.05–2.72)	2.98 (1.36–8.58)	-
NRTI	100%	-	100%	-
NNRTI	0%	-	14.2%	-
PI	28.5%	-	42.9%	-
II	42.9%	-	28.5%	-
Cocktail	57.1%	-	57.1%	-

HCV, hepatitis C virus; FIB-4, fibrosis-4; NRTI, nucleoside reverse transcriptase inhibitor; NNRTI, non-nucleoside reverse transcriptase inhibitor; PI, protease inhibitor; II, integrase inhibitor.

### iNKT cells are present in lower frequency and demonstrate impaired expansion to TCR agonism in the setting of HIV and HIV/HCV coinfection

**Figure 1:**
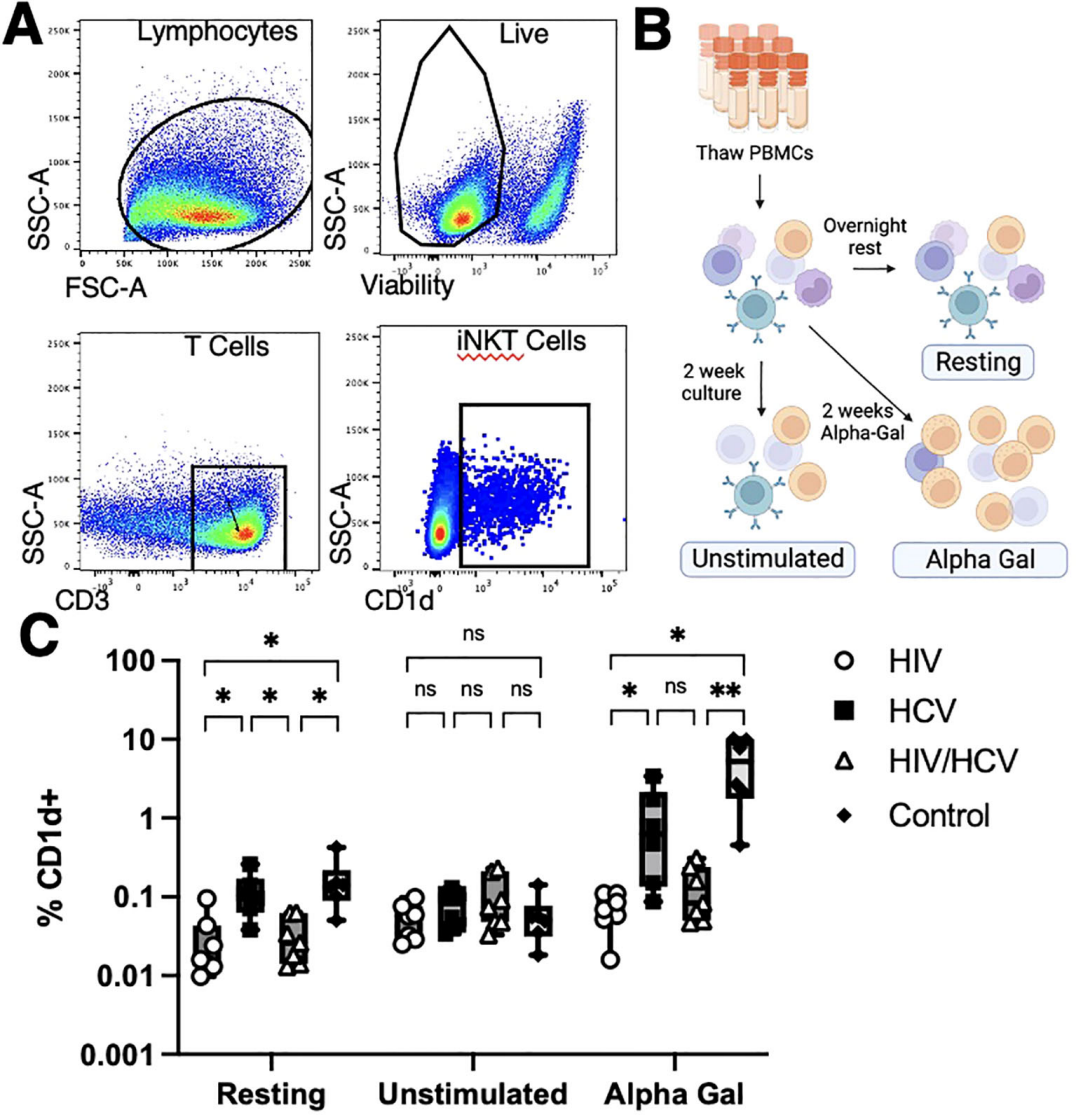
Methods overview and iNKT cell frequencies. **(A)** Identification of iNKT cells with CD1d dextramer loaded with alpha-gal. iNKT cells were identified as live T cells that bound the CD1d dextramer. **(B)** Patient samples were analyzed at fresh thaw (Resting), 2 weeks cultured with media alone (Unstimulated), or cultured 2 weeks in alpha-gal (Alpha-Gal). **(C)** iNKT cells from people living without HCV or HIV and people living with HCV monoinfection had higher resting frequencies and higher frequencies upon alpha-gal expansion than those from people living with HIV (Mann–Whitney U with Bonferroni correction, *p < 0.5, ** p < 0.01). iNKT, invariant natural killer T; alpha-gal, alpha-galactosylceramide; HCV, hepatitis C virus.

We assessed the frequency and phenotype of circulating iNKT in all participants. To identify the iNKT cells in the PBMC compartment, we utilized the strategy described in **Figure 1A** by gating on live and CD3+ cells that bound a CD1d-alpha-galceramine dextramer conjugated to PE. We assessed the frequency and differentiation stages of iNKT cells in three conditions: after overnight rest, after a 2-week culture with IL-2, and after a 2-week culture with IL-2 and alpha-gal, a TCR agonist for iNKT cells (**Figure 1B**). We observed that people with HIV and HIV/HCV had lower circulating frequencies of iNKT cells after overnight rest (**Figure 1C**). Moreover, the frequency of the iNKT cells in people with HIV and HIV/HCV only expanded an average of 1.6- and 2.4-fold, respectively (overall range 0.16–9-fold), after stimulation and *in vitro* culture compared to the 15.66- and 103-fold average, respectively (overall range 1.9–181-fold), observed in the people with HCV and the control group.

### iNKT cells from people with HIV, HCV, and HIV/HCV express distinct differentiation markers

**Figure 2:**
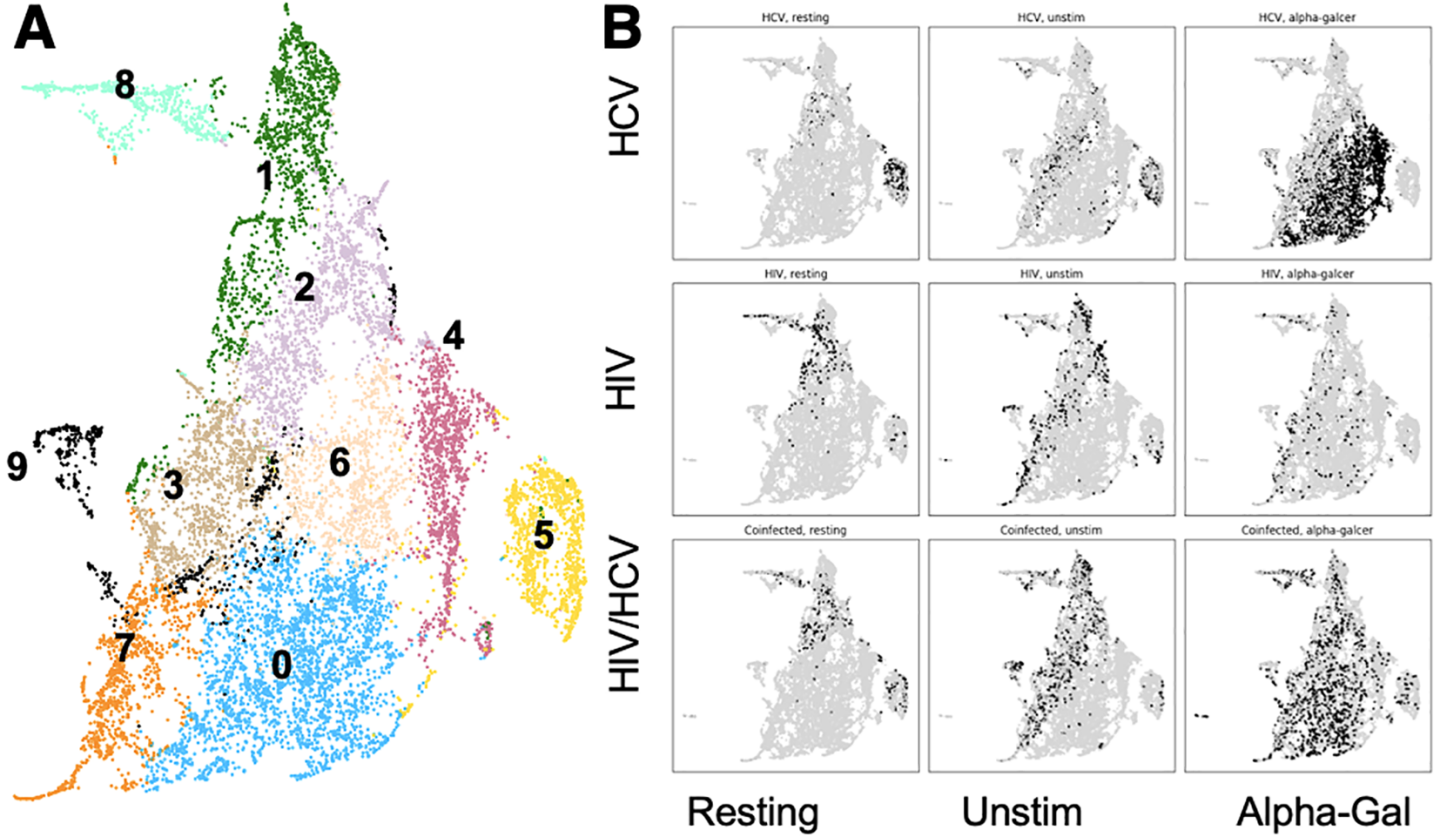
Cluster distribution of iNKT cells and cluster phenotypes. **(A)** UMAP with 14 different clusters of iNKT cells based on surface marker expression. **(B)** UMAP indicating the location of cells (black) from each combination of disease state and stimulation condition. iNKT, invariant natural killer T; UMAP, Uniform Manifold Approximation and Projection.

Focusing on only cells that bound CD1d, we used a Python-based flow cytometry analysis platform, FlowKit, to evaluate the phenotypes of cells from the three cohorts and three stimulation conditions with chronic infection based on the expression of the lineage, differentiation, and activation markers using unsupervised Leiden clustering. As reported with the UMAP in **Figure 2**, we identified 10 distinct clusters based on phenotypes of these cells (**Figure 2A**). After subdividing the clusters based on disease state, it became apparent that the cells of people from HCV clustered separately from those with HIV and HIV/HCV (**Figure 2B**). The evaluation of the distribution among stimulation conditions revealed that resting and unstimulated cells clustered separately from alpha-gal-stimulated cells in samples from people with HCV, while in samples from PWH, resting, unstimulated, and alpha-gal expanded iNKT cells clustered similarly (**Figure 2B**).

### iNKT cells from people with HIV and HIV/HCV are more likely to be CD8+ and CD57+ and have downregulated TCRs compared to those with HCV and controls

**Figure 3:**
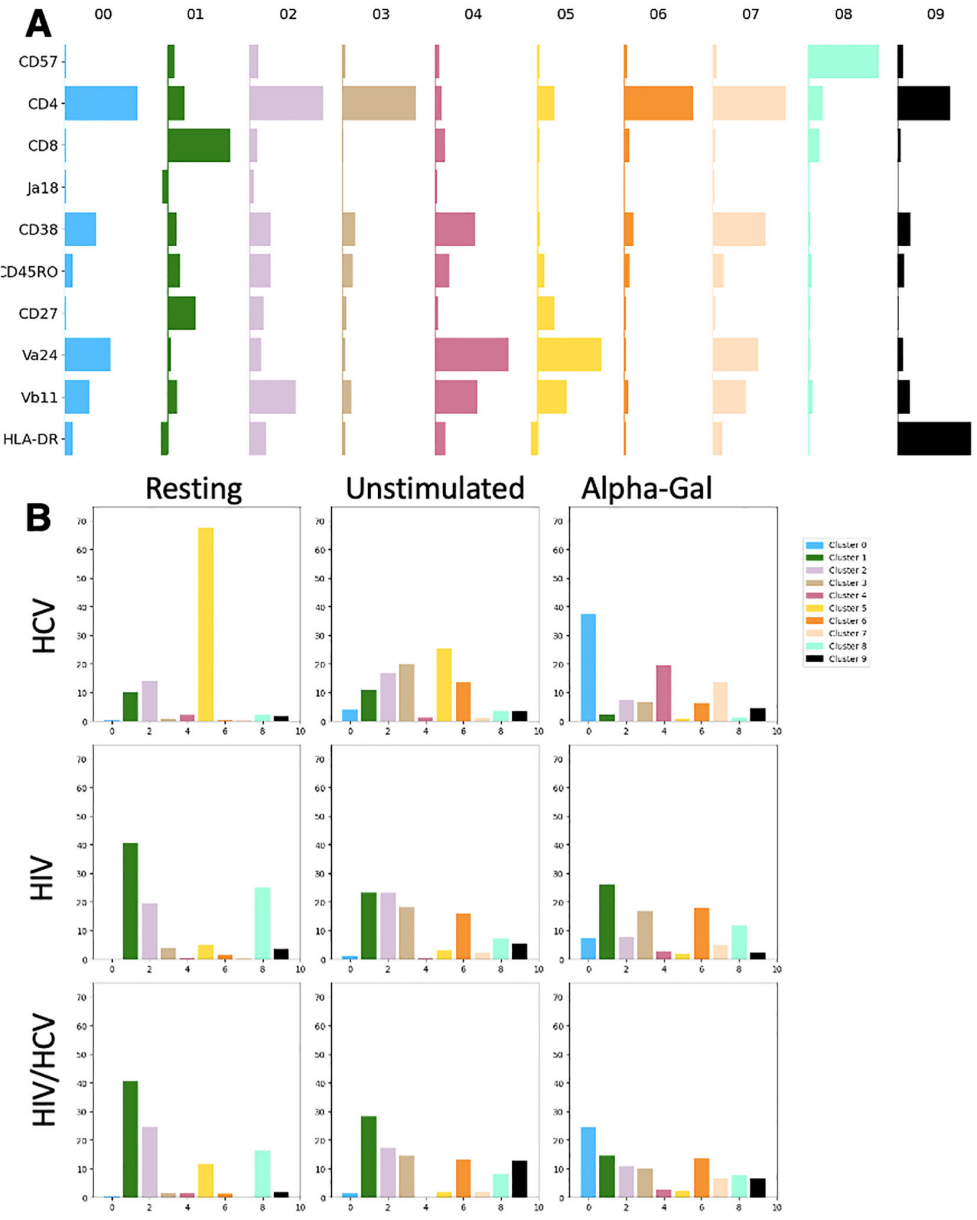
Expression level of markers by cluster and distribution of clusters among disease states and stimulation conditions. **(A)** Markers (rows) relative Median Fluorescence Intensity (MFI) (bar width) in each cluster (columns). Bar horizontal height is proportional to log MFI. **(B)** Percentage of iNKT cells in each combination disease state and stimulation condition belonging to each cluster. iNKT, invariant natural killer T; HCV, hepatitis C virus.

The relative expression levels of 10 markers selected to reflect maturation and activation state as well as TCR expression levels within each cluster are reported in **Figure 3A**. The CD4+ CD8− cells represent the early/naïve iNKT cells, double negative represents the effector, and CD8 single positive represents terminal differentiation. CD57 is a marker for exhaustion and terminal differentiation, and Vα24, Vβ11, and Vα24-Jα18 (Ja18) represent the individual TCR chains and the rearranged TCR. CD38 and HLA-DR are markers of activation, and CD27 in combination with CD45RO defines central memory (CD45RO+ CD27+), effector memory (CD45RO+ CD27−), and naïve/early (CD45RO− CD27+) sets for conventional T cells. We next investigated the distribution of phenotypes among disease states and stimulation conditions (**Figure 3B**). For HIV in the resting condition, 40.7% of the cells belonged to cluster 1, which was defined by CD8 single positivity. In contrast, for HCV in resting condition, the majority (67.5%) of cells were grouped in cluster 5, which was characterized by CD4 dim, CD8−. The HIV/HCV coinfection cohort in resting condition was much more similar to the HIV monoinfection cohort. In the HIV and HIV/HCV cohorts in the resting state, there was also a significant contribution of clusters 2 and 8. Cluster 8 was defined by very high CD57 expression, which was only minimally represented in HCV monoinfection. The unstimulated conditions between the three cohorts with chronic infection largely looked like their corresponding resting conditions but with the expansion of populations 3 and 6, which correspond to resting cells as well.

The most significant differences between cohorts were observed in the alpha-gal-stimulated condition. For people with HCV, there was a large shift in the distribution of cells from cluster 5 to clusters 0, 4, and 7 (37.2%, 19.5%, and 13.7%, respectively). Clusters 0 and 7 were phenotypically similar and were defined by CD38 upregulation, high expression of TCR components, and CD4 single positivity. Cluster 4 appeared to be a double-negative population, likely following the normal progression of activation and maturation as has been previously described (7, 8). These cells were behaving as the literature has suggested, with TCR agonism causing expansion (**Figure 1D**), activation, and progression through the normal maturation pathway. In contrast, in people with HIV monoinfection, the resting and activated conditions were nearly identical with their cluster contributions, indicating the minimal impact of the additional stimulus on surface marker expression. HIV/HCV looked like the additive effect of both infections, with phenotypes of each condition represented in the distribution of cells among clusters. Interestingly, the one exception to this was the lack of significant expansion of cluster 4, which represents the effector population, which iNKT cells in HIV and HIV/HCV did not seem to achieve.

### iNKT cells from people with HCV more closely resemble iNKT cells from controls than iNKT cells from people with HIV

**Figure 4:**
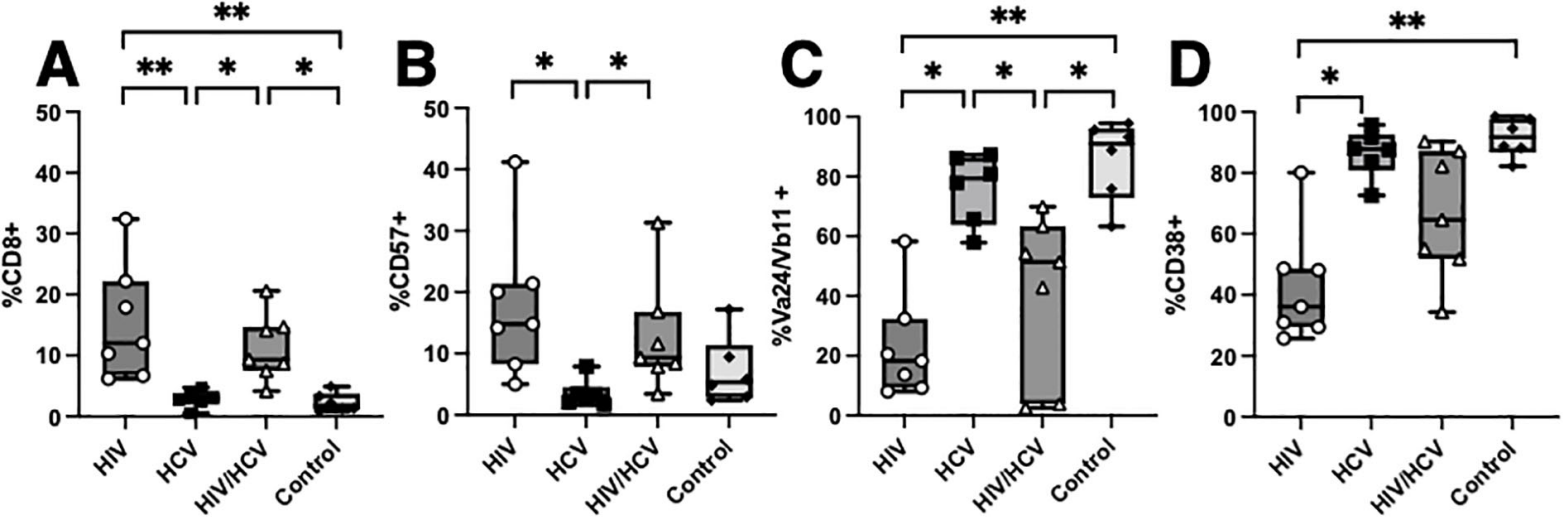
Comparison of HIV, HCV, and HIV/HCV phenotypes to controls. **(A–D)** Boolean gating of CD8, CD57, Va24/Vb11, and CD38 with comparison to controls in the alpha-gal-stimulated condition (Mann–Whitney U with Bonferroni correction, *p < 0.05, **p < 0.01). HCV, hepatitis C virus; alpha-gal, alpha-galactosylceramide.

Next, we aimed to compare the phenotypic differences observed from UMAP-based clustering to traditional binary gating and compare this to the phenotypes from controls. We focused on the alpha-gal-stimulated condition because we observed the largest phenotypic differences between groups after stimulation under these conditions, and the iNKT cells from the resting and unstimulated conditions from people with HIV were of very low frequency and thus had less sensitivity for analysis (**Figure 1C**). In accordance with the UMAP clusters, we saw that people with HIV and HIV/HCV had higher frequencies of terminally differentiated iNKT expressing CD8 (**Figure 4A**) and CD57 (**Figure 4B**). The cells from these individuals also showed lower levels of TCR components Va24 and Vb111, which suggests TCR downregulation following activation (**Figure 4C**), and lower frequencies of CD38+ cells upon activation (**Figure 4D**), which suggests an impaired activation profile and mirrors their limited expansion potential (**Figure 1C**). Taken together, these data suggest that compared to HCV monoinfection and controls, iNKT cells from people with HIV and HIV/HCV are more anergic to stimulation.

## Discussion

iNKT cells are a population of innate-like T cells with the ability to rapidly respond to pathogens and aid in tissue repair, yet the roles of these cells in viral disease progression are not yet fully understood. iNKT cells are traditionally difficult to study in humans given their low circulating frequency, and their role in HCV infection is even more difficult to elucidate given the limited animal models (9, 29, 30). Using clinically derived samples, we conducted an extensive unbiased analysis to assess the proliferative capability and differentiation and activation phenotypes of this cellular subset in people with chronic HIV and HCV infection, including coinfection with both viruses. We discovered that the circulating iNKT cells from people with HIV and HIV/HCV coinfection have poor expansion and activation abilities upon stimulation with alpha-gal and are phenotypically shifted toward surface markers associated with terminal differentiation and anergy. Taken together, our data suggest an anergic state due to HIV infection that may impair antiviral response or impede healing following liver injury. Our data complement our previous observations on the increased aging phenotype of the circulating iNKT cells in people with HIV and their potential for tissue repair (31). Future steps for this project will determine if these same differences hold true in the liver resident iNKT cells, as well as evaluate the impact of race, sex, order of infection, and antiviral regimen on these phenotypes and functions.

To examine the impact of HIV and HCV on iNKT phenotypes, we performed an extensive analysis through the surface expression of T-cell markers as well as their proliferative capacity to TCR agonism. In humans, the characterization of iNKT subpopulations in disease is incomplete, partially due to the low circulating frequency, and this study allowed for an unbiased approach to differentiate phenotypes using FlowKit, the Python-based approach to unbiased clustering (25). Moreover, FlowKit-based clustering can be tailored to the specification of each application as well as robust control over data representation and formatting. This study mainly aimed to compare HCV to HIV/HCV coinfection, and, although we were limited in our ability to include the healthy controls in this UMAP analysis due to the collection and analysis being performed several years later, we could still analyze the data to answer our initial question. We used samples collected from people living without infection to validate the findings of the UMAP-based method by applying traditional gating to the original flow data, which supported the notion of a shift toward anergy and senescence. The utilization of this software and the techniques described to phenotype rare cellular subsets provide a new path for future research in the field of chronic diseases. Overall, this study revealed an impaired proliferative profile of the iNKT subset that could be relevant for the design of immunotherapeutic approaches.The changes observed in iNKT phenotype and proliferative ability in people with HIV/HCV coinfection suggest an impairment that may be contributing to the enhanced pathogenesis during coinfection and could inform novel therapeutic approaches. While it is unknown if there are therapies that could reconstitute iNKT functionality, the concept of iNKT adoptive transfer has been widely explored in the context of cancer immunotherapy. iNKT cells and CD1d are monomorphic and have a low risk of graft-versus-host disease when adoptively transferred (32–34). Several groups have trialed expanding iNKT cells *ex vivo* using alpha-gal and reinfusing them as a treatment for cancer, especially hepatocellular carcinoma given their propensity to hone to the liver (2, 35). However, this approach appears to have been limited, as the CD4− and CD8+ iNKT cells are the more cytotoxic effector cells and are of lower frequency than the pro-repair CD4+ iNKT cells (2, 30). In the setting of liver injury in the setting of HCV infection, we would argue that the infusion and replacement of the lost CD4+ iNKT subset in people with HIV would be preferable and could be a therapy that may be able to aid in liver regeneration during antiviral treatment for HCV.

We observed differences in the phenotypic and proliferative capabilities of the four cohorts of participants that are novel and represent a key piece of knowledge in understanding how these cells contribute to viral disease. We believe that this study provides a new understanding of the alterations that the iNKT subset experiences in the setting of HIV, HCV, and HIV/HCV infections. A future direction of this study will be to compare these surface phenotypes with gene expression profiles using single-cell RNA sequencing to allow for pseudo time analysis of these populations to determine their maturation pathways within each clinical condition and in controls. We also need to determine to what extent these phenotypical changes correlate with the liver resident iNKT cells as well as with liver pathogenesis.

## Data Availability

The raw data supporting the conclusions of this article will be made available by the authors, without undue reservation.

## References

[1] Kinjo Y Takatsuka S Kitano N Kawakubo S Abe M Ueno K. Functions of CD1d-restricted invariant natural killer T cells in antimicrobial immunity and potential applications for infection control. Front Immunol. (2018) 9:1266. doi: 10.3389/fimmu.2018.01266 29928278 PMC5997780

[2] Look A Burns D Tews I Roghanian A Mansour S. Towards a better understanding of human iNKT cell subpopulations for improved clinical outcomes. Front Immunol. (2023) 14:1176724. doi: 10.3389/fimmu.2023.1176724 37153585 PMC10154573

[3] Porcelli SA Modlin RL. The CD1 system: antigen-presenting molecules for T cell recognition of lipids and glycolipids. Annu Rev Immunol. (1999) 17:297–329. doi: 10.1146/annurev.immunol.17.1.297 10358761

[4] Bleicher PA Balk SP Hagen SJ Blumberg RS Flotte TJ Terhorst C. Expression of murine CD1 on gastrointestinal epithelium. Science. (1990) 250(4981):679–82. doi: 10.1126/science.1700477 1700477

[5] Zhu S Zhang H Bai L. NKT cells in liver diseases. Front Med. (2018) 12(3):249–61. doi: 10.1007/s11684-018-0622-3 29623561

[6] Roederer M Quaye L Mangino M Beddall MH Mahnke Y Chattopadhyay P. The genetic architecture of the human immune system: a bioresource for autoimmunity and disease pathogenesis. Cell. (2015) 161(2):387–403. doi: 10.1016/j.cell.2015.02.046 25772697 PMC4393780

[7] Gumperz JE Miyake S Yamamura T Brenner MB. Functionally distinct subsets of CD1d-restricted natural killer T cells revealed by CD1d tetramer staining. J Exp Med. (2002) 195(5):625–36. doi: 10.1084/jem.20011786 PMC2193772 11877485

[8] Lee PT Benlagha K Teyton L Bendelac A. Distinct functional lineages of human Vα24 natural killer T cells. J Exp Med. (2002) 195(5):637–41. doi: 10.1084/jem.20011908 PMC2193771 11877486

[9] Guan J Wang G Yang Q Chen C Deng J Gu X. Natural killer T cells in various mouse models of hepatitis. BioMed Res Int. (2021) 2021:1782765. doi: 10.1155/2021/1782765 33506011 PMC7810568

[10] Senff T Menne C Cosmovici C Lewis-Ximenez LL Aneja J Broering R. Peripheral blood iNKT cell activation correlates with liver damage during acute hepatitis C. JCI Insight. (2022) 7(2):e155432. doi: 10.1172/jci.insight.155432 34905514 PMC8855829

[11] Raus S Lopez-Scarim J Luthy J Billerbeck E. Hepatic iNKT cells produce type 2 cytokines and restrain antiviral T cells during acute hepacivirus infection. Front Immunol. (2022) 13:953151. doi: 10.3389/fimmu.2022.953151 36159876 PMC9501689

[12] Miyaki E Hiraga N Imamura M Uchida T Kan H Tsuge M. Interferon alpha treatment stimulates interferon gamma expression in type i NKT cells and enhances their antiviral effect against hepatitis C virus. PloS One. (2017) 12(3):e0172412. doi: 10.1371/journal.pone.0172412 28253324 PMC5333814

[13] Bandyopadhyay K Marrero I Kumar V. NKT cell subsets as key participants in liver physiology and pathology. Cell Mol Immunol. (2016) 13(3):337–46. doi: 10.1038/cmi.2015.115 PMC4856801 26972772

[14] Crosby CM Kronenberg M. Tissue-specific functions of invariant natural killer T cells. Nat Rev Immunol. (2018) 18(9):559–74. doi: 10.1038/s41577-018-0034-2 PMC6343475 29967365

[15] Harada K Isse K Tsuneyama K Ohta H Nakanuma Y. Accumulating CD57 + CD3 + natural killer T cells are related to intrahepatic bile duct lesions in primary biliary cirrhosis. Liver Int. (2003) 23(2):94–100. doi: 10.1034/j.1600-0676.2003.00807.x 12654131

[16] Syn WK Oo YH Pereira TA Karaca GF Jung Y Omenetti A. Accumulation of natural killer T cells in progressive nonalcoholic fatty liver disease. Hepatology. (2010) 51(6):1998–2007. doi: 10.1002/hep.23599 20512988 PMC2920131

[17] Huang W He W Shi X He X Dou L Gao Y. The role of CD1d and MR1 restricted T cells in the liver. Front Immunol. (2018) 9:2424. doi: 10.3389/fimmu.2018.02424 30425710 PMC6218621

[18] Moll M Kuylenstierna C Gonzalez VD Andersson SK Bosnjak L Sönnerborg A. Severe functional impairment and elevated PD-1 expression in CD1d-restricted NKT cells retained during chronic HIV-1 infection. Eur J Immunol. (2009) 39(3):902–11. doi: 10.1002/eji.200838780 PMC2736548 19197939

[19] Vasan S Poles MA Horowitz A Siladji EE Markowitz M Tsuji M. Function of NKT cells, potential anti-HIV effector cells, are improved by beginning HAART during acute HIV-1 infection. Int Immunol. (2007) 19(8):943–51. doi: 10.1093/intimm/dxm055 17702988

[20] Li D Xu XN. NKT cells in HIV-1 infection. Cell Res. (2008) 18(8):817–22. doi: 10.1038/cr.2008.85 18645582

[21] Paquin-Proulx D Lal KG Phuang-Ngern Y Creegan M Tokarev A Suhkumvittaya S. Preferential and persistent impact of acute HIV-1 infection on CD4^+^ iNKT cells in colonic mucosa. Proc Natl Acad Sci USA. (2021) 118(46):e2104721118. doi: 10.1073/pnas.2104721118 34753817 PMC8609642

[22] Smith CJ Ryom L Weber R Morlat P Pradier C Reiss P. D:A:D study group. trends in underlying causes of death in people with HIV from 1999 to 2011 (D:A:D): a multicohort collaboration. Lancet. (2014) 384(9939):241–8. doi: 10.1016/S0140-6736(14)60604-8 25042234

[23] Thein HH Yi Q Dore GJ Krahn MD. Natural history of hepatitis C virus infection in HIV-infected individuals and the impact of HIV in the era of highly active antiretroviral therapy: a meta-analysis. AIDS. (2008) 22(15):1979–91. doi: 10.1097/QAD.0b013e32830e6d51 18784461

[24] Konerman MA Mehta SH Sutcliffe CG Vu T Higgins Y Torbenson MS. Fibrosis progression in human immunodeficiency virus/hepatitis C virus coinfected adults: prospective analysis of 435 liver biopsy pairs. Hepatology. (2014) 59(3):767–75. doi: 10.1002/hep.26741 PMC3943751 24436062

[25] White S Quinn J Enzor J Staats J Mosier SM Almarode J. FlowKit: A python toolkit for integrated manual and automated cytometry analysis workflows. Front Immunol. (2021) 12:768541. doi: 10.3389/fimmu.2021.768541 34804056 PMC8602902

[26] McInnes L Healy J Saul N Großberger L. UMAP: Uniform manifold approximation and projection. J Open Source Softw. (2018) 3. doi: 10.21105/joss.00861

[27] Traag VA Waltman L van Eck NJ. From Louvain to Leiden: guaranteeing well-connected communities. Sci Rep. (2019) 9(1):5233. doi: 10.1038/s41598-019-41695-z 30914743 PMC6435756

[28] Levine JH Simonds EF Bendall SC Davis KL Amir E-a D Tadmor MD. Data-driven phenotypic dissection of AML reveals progenitor-like cells that correlate with prognosis. Cell. (2015) 162(1):184–97. doi: 10.1016/j.cell.2015.05.047 PMC4508757 26095251

[29] Guan J Wang G Yang Q Chen C Deng J Gu X. Natural killer T cells in various mouse models of hepatitis. BioMed Res Int. (2021) 2021:1782765. doi: 10.1155/2021/1782765 33506011 PMC7810568

[30] Hadiloo K Tahmasebi S Esmaeilzadeh A. CAR-NKT cell therapy: a new promising paradigm of cancer immunotherapy. Cancer Cell Int. (2023) 23(1):86. doi: 10.1186/s12935-023-02923-9 37158883 PMC10165596

[31] Naggie S Swiderska-Syn M Choi S Lusk S Lan A Ferrari G. Markers of tissue repair and cellular aging are increased in the liver tissue of patients with HIV infection regardless of presence of HCV coinfection. Open Forum Infect Dis. (2018) 5(7):ofy138. doi: 10.1093/ofid/ofy138 29992177 PMC6030967

[32] Rubio MT Bouillié M Bouazza N Coman T Trebeden-Nègre H Gomez A. Pre-transplant donor CD4^-^ invariant NKT cell expansion capacity predicts the occurrence of acute graft-versus-host disease. Leukemia. (2017) 31(4):903–12. doi: 10.1038/leu.2016.281 27740636

[33] Chaidos A Patterson S Szydlo R Chaudhry MS Dazzi F Kanfer E. Graft invariant natural killer t-cell dose predicts risk of acute graft-versus-host disease in allogeneic hematopoietic stem cell transplantation. Blood. (2012) 119(21):5030–6. doi: 10.1182/blood-2011-11-389304 PMC6143158 22371885

[34] Rotolo A Caputo VS Holubova M Baxan N Dubois O Chaudhry MS. Enhanced anti-lymphoma activity of CAR19-iNKT cells underpinned by dual CD19 and CD1d targeting. Cancer Cell. (2018) 34(4):596–610.e11. doi: 10.1016/j.ccell.2018.08.017 30300581 PMC6179961

[35] Gao Y Guo J Bao X Xiong F Ma Y Tan B. Adoptive transfer of autologous invariant natural killer T cells as immunotherapy for advanced hepatocellular carcinoma: A phase i clinical trial. Oncologist. (2021) 26(11):e1919–30. doi: 10.1002/onco.13899 PMC8571770 34255901

